# Association between immune checkpoint inhibitors and myocardial infarction in Asians: A population‐based self‐controlled case series

**DOI:** 10.1002/cam4.5729

**Published:** 2023-02-21

**Authors:** Jeffrey Shi Kai Chan, Pias Tang, Teddy Tai Loy Lee, Oscar Hou In Chou, Yan Hiu Athena Lee, Guoliang Li, Fung Ping Leung, Wing Tak Wong, Tong Liu, Gary Tse

**Affiliations:** ^1^ Cardio‐Oncology Research Unit Cardiovascular Analytics Group, UK‐China Collaboration Hong Kong China; ^2^ Department of Emergency Medicine, Li Ka Shing Faculty of Medicine University of Hong Kong Hong Kong China; ^3^ Division of Clinical Pharmacology, Department of Medicine, School of Clinical Medicine, Li Ka Shing Faculty of Medicine University of Hong Kong Hong Kong China; ^4^ Department of Cardiovascular Medicine The First Affiliated Hospital of Xi'an Jiaotong University Xi'an China; ^5^ School of Life Sciences The Chinese University of Hong Kong Hong Kong China; ^6^ Tianjin Key Laboratory of Ionic‐Molecular Function of Cardiovascular Disease, Department of Cardiology Tianjin Institute of Cardiology, Second Hospital of Tianjin Medical University Tianjin China; ^7^ Kent and Medway Medical School University of Kent and Canterbury Christ Church University Canterbury UK; ^8^ Department of Health Sciences, School of Nursing and Health Studies Hong Kong Metropolitan University Hong Kong China

**Keywords:** cancer management, check point control, clinical cancer research, epidemiology

## Abstract

**Background:**

While immune checkpoint inhibitors (ICIs) are associated with elevated cardiovascular risks, evidence of any association between ICIs and myocardial infarction (MI) was scarce, especially in Asians.

**Methods:**

Using prospectively collected population‐based data, this self‐controlled case series included patients prescribed an ICI between 1/1/2014 and 31/12/2020 in Hong Kong who had MI within January 1, 2013 to December 31, 2021. Incidence rate ratios (IRRs) for MI during and after ICI exposure were estimated, compared to the year before ICI initiation.

**Results:**

Of 3684 identified ICI users, 24 had MI during the study period. MI incidence increased significantly in the first 90 days of exposure (IRR 3.59 [95% confidence interval: 1.31–9.83], *p* = 0.013), but not days 91–180 (*p* = 0.148) or ≥181 (*p* = 0.591) of exposure, nor postexposure (*p* = 0.923). Sensitivity analyses excluding patients with MI‐related death and incorporating extended exposure periods produced consistent results separately.

**Conclusions:**

ICIs were associated with increased MI incidence in Asian Chinese patients during the first 90 days of use, but not later.

## Graphical abstract

Using prospectively collected population‐based data, this self‐controlled case series investigated the link between immune checkpoint inhibitors (ICIs) use and myocardial infarction (MI) in Chinese patients. Compared to the year prior to ICI use, MI incidence increased significantly in the first 90 days of exposure, but not in later exposure periods nor post‐exposure.

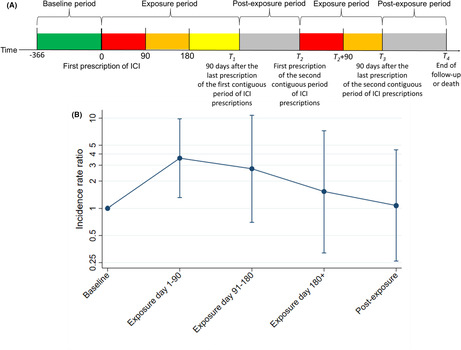

## 1 INTRODUCTION

Immune checkpoint inhibitors (ICIs) have become a common treatment for many types of cancer. A previous study suggested that ICIs may be associated with atherosclerosis and myocardial infarction (MI).^1^ However, unlike ICI‐related myocarditis which was relatively well‐characterized,^2^ evidence for ICI‐related MI had remained scarce, especially in Asians. With evidence demonstrating racial disparities in the presence and severity of coronary atherosclerosis,^3^ as well as the incidence and outcome ICI‐related adverse events,^4^ associations between ICIs and MI in Asians warrant further investigations. This study thus explored such associations in Asians.

## 2 MATERIALS AND METHODS

This study was approved by The Joint Chinese University of Hong Kong–New Territories East Cluster Clinical Research Ethics Committee and adhered with the Declaration of Helsinki. Patient consent was waived as deidentified data were used. Data were extracted from the Clinical Data Analysis and Reporting System, a prospective, population‐based database of patients attending public healthcare facilities in Hong Kong with linked mortality data. This system has been used in research with demonstrable data accuracy and completeness.^5^, ^6^, ^7^ All underlying data are available on reasonable request to the corresponding author.

This is a self‐controlled case series (SCCS). SCCS is a type of case‐only study in which only patients experiencing an outcome of interest are analyzed. It was chosen because, in an observational setting, it is difficult to identify appropriate control groups to be compared against patients receiving ICIs without incurring significant bias by indication, which arises when clinical differences between exposure groups drive both the exposure and the outcome. Bias by indication is notoriously difficult to address, and statistical adjustments are often inadequate.^8^ In SCCS, rather than using between‐individual analyses as in cohort studies, within‐individual comparisons of the incidence rate of events before and after exposure are performed. As each patient is compared with his/herself, all measured and unmeasured time‐invariant confounders are controlled for.^8^ The paired nature of the analysis used in SCCS also helps maintaining statistical power, thus allowing SCCS to be used reliably for relatively rare outcomes.^9^

**FIGURE 1:**
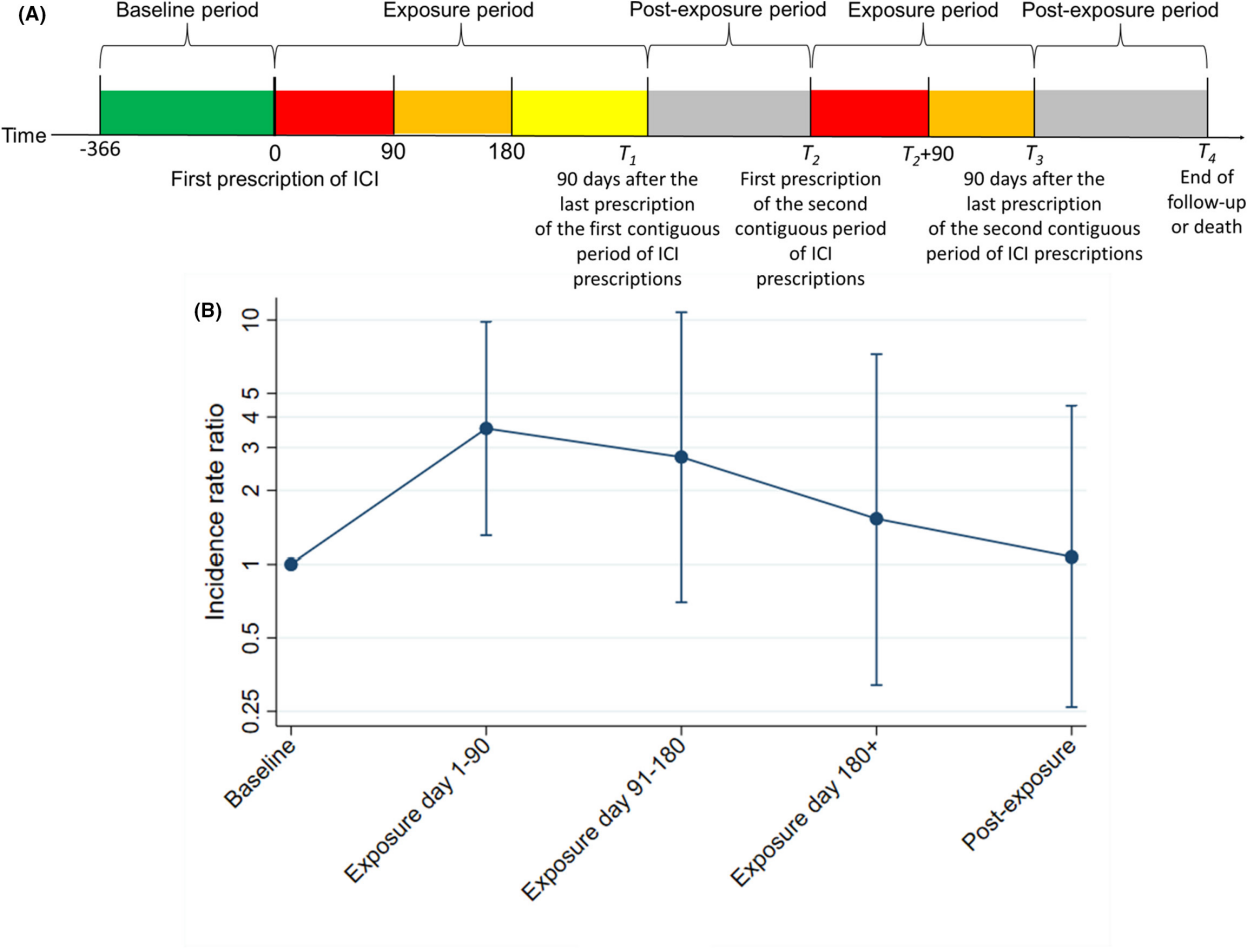
(A) Diagram illustrating the study design. Each analyzed subperiod of exposure is coded in a different color (red/orange/yellow). The second contiguous period of immune checkpoint inhibitor (ICI) prescriptions was shorter than 180 days, hence, the truncation of the 90–180 days subperiod of exposure (orange). (B) Incidence rate ratios for each subperiod of exposure and the postexposure period, compared to baseline.

Patients with cancer receiving any ICI (programmed cell death protein‐1 inhibitors [PD1i], PD ligand‐1 inhibitors [PDL1i], or cytotoxic T‐lymphocyte associated protein‐4 inhibitors [CTLA4i]) in Hong Kong between January 1, 2014 and December 31, 2020 were identified. Those without MI (identified using International Classification of Diseases, Ninth revision [ICD‐9] codes [410–411.0 and 412]) within January 1, 2013 to December 31, 2021 were only analyzed for the crude cohort‐level incidence rates (IRs), but not the SCCS analysis. The baseline period was defined as the year before the first ICI prescription. The exposure periods included contiguous ICI prescriptions (i.e., inter‐prescription gaps <60 days) and the ensuing 90 days, beyond which cardiac immune‐related adverse events were rare.^10^ Postexposure periods encompassed periods not described above, until death or end of follow‐up (December 31, 2021), whichever earlier. MI episodes over contiguous days were treated as singular events. Cardiovascular mortality within 30 days post‐event were deemed MI‐related.^11^ ICD codes used to identify cancer, cardiovascular mortality, and cardiovascular risk factors have been described elsewhere.^7^ The study design is summarized in Figure 1A.

Crude cohort‐level IRs pre‐ and post‐ICI initiation were calculated among all identified ICI users. SCCS analyses were performed on the final cohort using fixed‐effects conditional Poisson regression, with durations of the above subperiods as the exposure variable. Summary statistics were incidence rate ratios (IRRs) and 95% confidence intervals.

To account for the small but potentially important possibility of delayed cardiac events, an a priori sensitivity analysis was performed with the exposure period extended to 180 days after the last prescription within a contiguous prescription period. Additionally, as mortality associated with MI may skew estimates, another a priori sensitivity analysis was performed with exclusion of patients who had MI‐related mortality.

Furthermore, to minimize heterogeneity in the ICI used, a post hoc analysis was performed, restricting the analysis to those who only received PD1i. Due to small sample sizes, this analysis could neither be performed for PDL1i nor CTLA4i.

Two‐sided *p* < 0.05 were considered significant. All analyses were performed in Stata v16.1 (StataCorp LLC, USA).

## 3 RESULTS

Altogether, 3684 ICI users were identified (median follow‐up 442 [interquartile range: 145–989] days; median exposure 164 [91–315] days), of whom 24 had MI during the study period (20 PD1i users, one PDL1i user, and three PD1i + CTLA4i users; median follow‐up 436 [156–888] days; median exposure: 175.5 [115–412] days). Lung cancer occurred in nine, liver cancer in four, renal cancer in three, and other cancers in eight patients. Baseline demographic and cardiovascular risk factors are summarized in Table 1. Eight had MI during baseline (one PD1i user, three PD1i + CTLA4i users, and one PDL1i user), 12 during exposure (11 PD1i users and one PD1i + CTLA4i user), and four during postexposure (all PD1i users). Three had MI‐related death: two during exposure and one during postexposure. Crude cohort‐level MI IRs during the baseline, exposure, and postexposure periods were 2.2, 4.8, and 1.0 per 1000‐person‐years, respectively.

**TABLE 1:** Summary of baseline demographics and cardiovascular risk factors of the included patients.

Age, years	70 [60–77]^a^
Male sex, *N* (%)	17 (70.8)
Hypertension, *N* (%)	16 (66.7)
Diabetes mellitus, *N* (%)	9 (37.5)
Hyperlipidemia, *N* (%)	16 (66.7)
Ischemic heart disease, *N* (%)	11 (45.8)
Heart failure, *N* (%)	6 (25.0)
Chronic kidney disease, *N* (%)	1 (4.2)
Atrial fibrillation, *N* (%)	2 (8.3)
Stroke, *N* (%)	3 (12.5)

^a^ Median and interquartile range.

Compared to baseline, the incidence of MI was significantly higher in the first 90 days of exposure (IRR 3.59 [95% confidence interval 1.31–9.83], *p* = 0.013; Figure 1B), but not days 91–180 (IRR 2.74 [0.70–10.76], *p* = 0.148) or days ≥181 (IRR 1.53 [0.32–7.24], *p* = 0.591) of exposure, nor the postexposure period (IRR 1.07 [0.26–4.45], *p* = 0.923).

Consistently, in the sensitivity analysis with extended exposure period, the incidence of MI was significantly higher within the first 90 days of exposure (IRR 3.53 [1.29–9.67], *p* = 0.014), but not days 91–180 (IRR 2.01 [0.52–7.75], *p* = 0.309) or days ≥181 (IRR 1.13 [0.25–5.01], *p* = 0.884) of exposure, nor the postexposure period (IRR 1.94 [0.39–9.60], *p* = 0.418). The sensitivity analysis excluding the three patients who had MI‐related mortality also showed similar results, with a significantly higher MI incidence within the first 90 days of exposure (IRR 3.00 [1.05–8.59], *p* = 0.041), but not days 91–180 (IRR 1.61 [0.33–7.75], *p* = 0.556) or days ≥181 (IRR 0.83 [0.15–5.01], *p* = 0.838) of exposure, nor the postexposure period (IRR 0.89 [0.22–3.59], *p* = 0.868).

In the post hoc analysis of the 20 patients who only received PD1i, similar results were observed, with the incidence of MI being significantly higher within the first 90 days of exposure (IRR 5.21 [1.60, 17.06], *p* = 0.006), but not days 91–180 (IRR 3.60 [0.86, 15.08], *p* = 0.079) or days ≥181 (IRR 1.39 [0.33, 5.82], *p* = 0.651) of exposure, nor the postexposure period (IRR 0.87 [0.23, 3.24], *p* = 0.837).

## 4 DISCUSSION

To the best of our knowledge, this was the first SCCS exploring associations between ICIs and MI in Asians. The incidence of MI increased significantly within 90 days of ICI initiation but did not persist beyond this time period. Mechanistically, the early spike in MI incidence observed in this study agreed with previous animal studies in which short‐term ICI administration induced atherosclerotic plaque inflammation and progression.^12^ Specifically, accelerated atherosclerosis, vasculitis, and focal myocarditis mediated by ICI‐induced immune activation and inflammation have been proposed as likely mechanisms underlying ICI‐related MI, although other contributors such as sociodemographic factors and comorbidities may be at play too.^13^ In this cohort, cardiovascular risk factors were common which may have amplified the effects of ICI‐induced acceleration in atherosclerotic progression. Furthermore, we confirmed and extended previous clinical findings reporting increased MI incidence after ICI use,^1^ with the low IR of MI also comparable with prior observations.^1^, ^7^, ^10^ In particular, while previous clinical studies focused on the first event and neglected recurrent events,^1^ we considered recurrent events and provided novel evidence for the timing of spikes in the incidence of MI, echoing previous findings that most ICI‐related cardiovascular events occur early.^10^ Importantly, the postexposure MI incidence was not significantly different from baseline. This bridges an important gap in the literature, as previous investigations had not reported temporal variations in the risk of MI after ICI use, and, as chronic immune‐related adverse events become an increasing concern, it was unclear how long the cardiovascular risks associated with ICI use would persist.^14^

Using population‐based data, our findings were representative and likely generalizable to other Asian/Chinese cohorts. Clinically, these findings highlighted the importance of cardiac monitoring within the first 90 days of ICI use, which, according to the 2022 European Society of Cardiology guidelines, should be multidisciplinary.^15^ The postexposure normalization of MI incidence may reassure clinicians and patients over potential concerns for sustained increases in MI incidence, facilitating shared decision‐making. Moving forward, these novel data on the timing of MI incidence spiking should prompt further, larger investigations of the timing of cardiovascular risks to allow more granular recommendations for the scheduling of cardiac monitoring and follow‐up, as the current recommendations in this regard have inadequate levels of evidence (mostly level C only, i.e., from “consensus of opinion of the experts and/or small studies, retrospective studies, registries”).^15^ Furthermore, as it has been suggested that different classes of ICI may be associated with different cardiovascular risks,^16^ further studies should delineate class‐/agent‐specific associations with MI, as we did with patients who received only PD1i. Predictors and prognosticators of ICI‐related MI also warrant further investigations, as well as relevant treatments and prophylactic cardioprotective strategies—in particular, statins have been explored for the latter and have shown promising results, slowing atherosclerotic progression in ICI users and thus having the potential to prevent ICI‐related MI.^13^, ^17^

Nonetheless, this study was not devoid of limitations. It was limited by potential time‐varying confounders, event‐dependence of risks, and the presence of event‐related censoring. We attempted to mitigate event‐related censoring using a sensitivity analysis which excluded patients who had MI‐related mortality. This yielded consistent results, reinforcing our findings' validity. Also, data for MI subtypes were not available, and outcome adjudication was not possible due to the nature of the database. Nonetheless, this database has been demonstrated to have good data accuracy and completeness,^6^ and the small number of events observed was unlikely to have allowed further meaningful analysis of different MI subtypes. Overall, further studies are required to confirm our findings, as well as evaluating the generalizability of our findings to different populations.

In conclusion, ICIs were associated with increased MI incidence in Asian Chinese patients during the first 90 days of use, but not later.

## AUTHOR CONTRIBUTIONS

**Jeffrey Shi Kai Chan:** Conceptualization (equal); data curation (lead); formal analysis (lead); methodology (lead); project administration (equal); visualization (lead); writing – original draft (lead). **Pias Tang:** Conceptualization (equal); writing – review and editing (supporting). **Teddy Tai Loy Lee:** Investigation (equal); writing – review and editing (supporting). **Oscar Chou:** Investigation (equal); writing – review and editing (supporting). **Yan Hiu Athena Lee:** Investigation (equal); writing – review and editing (supporting). **Guoliang Li:** Supervision (supporting); writing – review and editing (supporting). **Fung Ping Leung:** Supervision (supporting); writing – review and editing (supporting). **Wing Tak Wong:** Supervision (equal); writing – review and editing (supporting). **Tong Liu:** Funding acquisition (lead); supervision (equal); writing – review and editing (supporting). **Gary Tse:** Investigation (equal); project administration (equal); resources (lead); writing – review and editing (equal).

## FUNDING INFORMATION

This work was supported by the Tianjin Key Medical Discipline (Specialty) Construction Project (Project number: TJYXZDXK‐029A).

## CONFLICT OF INTEREST STATEMENT

None of the authors have any conflict of interest to report.

## ETHICS STATEMENT

This study was approved by The Joint Chinese University of Hong Kong–New Territories East Cluster Clinical Research Ethics Committee and adhered with the Declaration of Helsinki. Patient consent was waived as deidentified retrospective data were used.

## Data Availability

All underlying data are available on reasonable request to the corresponding author.
